# Editorial: Autoantibodies in Kidney Diseases

**DOI:** 10.3389/fimmu.2020.591338

**Published:** 2020-09-16

**Authors:** Kevin J. Marchbank, Ashley Frazer-Abel, Marie-Agnes Dragon-Durey, Bradley P. Dixon

**Affiliations:** ^1^Translational and Clinical Research Institute, Newcastle University, Newcastle upon Tyne, United Kingdom; ^2^National Renal Complement Therapeutics Centre, Newcastle upon Tyne, United Kingdom; ^3^Medicine-Rheumatology, University of Colorado Denver, Denver, CO, United States; ^4^Laboratory of Immunology, Georges Pompidou European Hospital, APHP, INSERM UMRS1138, Cordeliers Research Center, Université de Paris, Paris, France; ^5^Department of Pediatric Nephrology, University of Colorado Denver, Denver, CO, United States; ^6^Department of Pediatric Nephrology, Children's Hospital Colorado, Aurora, CO, United States

**Keywords:** autoantibody (aAb), complement, aHUS, C3G, lupus, immune complex, IgAN pathogenesis, nephrology

The purpose of this Frontier in Immunology Research Topic. Autoantibodies (aAb) in Kidney Diseases was to bring diverse knowledge, experience and view-points together into one collection highlighting the role of aAb to complement proteins in the genesis and severity of kidney disease, and providing evidence for common pathways that lead to autoantibody-induced pathology and the emergence of the aAb themselves. It was postulated that deepened insight of the crosstalk between complement and autoantibody-based pathologies would provide unique opportunities to understand and treat these diseases. Reading this collection as a body of work should stimulate vivid conversations between you and your colleagues around new avenues of investigation.

At time of writing, greater than 30 thousand views of the articles in the collection has occurred, suggesting that one key aim has already been met; the dissemination of collated thoughts and key original research on this topic. The association of anti-Complement aAb with disease has been long appreciated, yet our understanding of their roles in development of disease still remains open to interpretation. Herein, and since the days they were called immunoconglutinins (see Vasilev et al.), understanding the linkage between nephritic factors (antibodies that bind the C3/C5 convertases) or the individual components of these complexes and their modification of disease processes is as important now (see the review by Hauer et al. and Corvillo et al.) as it ever was. Our collection provides considered perspective on the mechanisms of action of nephritic factors at various stages of the complement pathway (see Hauer et al. Corvillo et al. and Zhao et al.), with different consequences/outcomes depending on titer, subclass of Ig and context. Importantly, the precision of current testing to characterize nephritic factors is also key from a clinical perspective, beautifully appraised by Corvillo et al.. Zhao et al. also reminds us that the *CFHR* gene deletions that predominately associate with anti-FH formation in aHUS do not co-associate with C3 nephritic factors in C3G and speaks to multiple routes to the generation of aAb in kidney diseases. Thus, the articles herein also provide insight into the evolution of anti-complement protein/convertase aAb by explaining the potential roles of the cryptic epitope, altered self and immune “danger signal” that undoubtedly interact with patient genetic factors to deliver the perfect storm of events that generate aAb; to generate the immune complexes (IC) or non-lethal membrane attack complex (MAC) that wreak havoc in the kidney.

Collectively, we have learned that much still remains to be discovered from anti-FH aAb associated with aHUS. Puraswani et al. noted in their large longitudinal cohort study that high anti-FH titers predict relapse, but do not correlate well with disease activity. The establishment of an international reference titer has allowed a degree of correlation between high titers (over 1,000 AU/RU) and low levels of C3, confirming activation of the alternative pathway. However, correlation between low free-FH levels, predicting early relapses in patients, with high anti-FH titers as described by Puraswani et al. is less common in other studies. It likely underlines that immune complexed FH is not well-tolerated, similar to anti-dsDNA (Wang and Xia). “Stable” autoantibody titer over time appears both historically and contemporaneously a common feature of anti-complement protein aAb in many patients across the reviews and primary research in this collection.

Another major commonality is the importance of prompt detection of aAb followed by rapid delivery of clinical intervention to achieve optimal outcomes, eculizumab being particularly effective in many studies despite not altering aAb titer. Interestingly, Zhao et al. also outlined the potential for eculizumab to halt complement dysregulation at the level of C5 in the presence of C5 nephritic factors, again suggesting rationale for its use where high tire anti-complement aAb are identified early in the disease course. Through the use of highly defined control populations, Valoti et al. re-affirmed the genetic and environmental aspects of the development of anti-FH aAb clarifying the complexity of both the genesis and patho-mechanistic outcomes associated with anti-FH aAb. Wang and Xia provide a stellar review of anti-dsDNA aAbs allowing a side by side comparison of the commonality and key differences noted to anti-complement aAbs. For instance, we are reminded that antigen presentation pathways and loss of tolerance are underappreciated in the genesis of anti-complement protein aAb, and that similar mechanisms must lie behind breaks in tolerance across these diseases.

AAb targeting to cell surfaces in the kidney generates multifaceted immune activation through direct stimulation of cells as is highlighted in the review of autoimmune membranous nephritis by Liu et al. Activation of cells through IC deposition, both directly and indirectly via multiple signaling pathways, exemplifies the challenges that are faced in deciphering the mechanisms that drive kidney disease. Non-lethal effects of complement activation are a significant factor in endothelial, epithelial, podocyte, and tubulointerstitial injury in the kidney. This is further confirmed through the study by Goutaudier et al. focused on C5b-9 as a biomarker of poor prognosis in the context of antibody mediated rejection, suggesting that assembly of MAC on renal tissues, understandably, is a marker for the more severe cases of Ab mediated rejection, ineffective complement regulation and potentially a driver of GBM structural changes. Rizk et al. present a clear and compelling case for control of complement hyperactivity in IgA Nephropathy (IgAN). Historically it is well-appreciated that C3 deposition is extremely common in the glomeruli of patients with IgAN and our knowledge of the autoimmune process in IgAN is arguably the most developed. Altered self, driving auto-immunity–epitope drift, failure to correctly solubilize or clear the resultant IC systemically, and a failure to clear deposited IC in the kidney due to continued load drives AP activation and C3 deposition in the glomerular mesangium in a vicious cycle similar to other IC-driven disease discussed here. All in all, compelling evidence is given that restoration of the finely balanced control of the AP and LP in IgAN may have benefit for many patients, and together with most diseases discussed herein, with the number of complement-directed drugs coming to the clinic, targeted options for the treatment of these diseases may soon be at hand.

## Author Contributions

KM wrote the initial draft of the editorial, this was reviewed principally by BD with comments from AF-A and M-AD-D. All authors contributed to the article and approved the submitted version.

## Conflict of Interest

The authors declare that the research was conducted in the absence of any commercial or financial relationships that could be construed as a potential conflict of interest.

